# Variability in German Cockroach Extract Composition Greatly Impacts T Cell Potency in Cockroach-Allergic Donors

**DOI:** 10.3389/fimmu.2019.00313

**Published:** 2019-02-27

**Authors:** Giovanni Birrueta, April Frazier, Anna Pomés, Jill Glesner, Stephanie Filep, Coby Schal, Kyoung Yong Jeong, Curtis McMurtrey, Thomas Vander Schans, William H. Hildebrand, Paula Busse, Avraham Beigelman, Leonard B. Bacharier, Bjoern Peters, Alessandro Sette, Véronique Schulten

**Affiliations:** ^1^La Jolla Institute for Immunology, La Jolla, CA, United States; ^2^Indoor Biotechnologies, Inc., Charlottesville, VA, United States; ^3^Department of Entomology and Plant Pathology, North Carolina State University, Raleigh, NC, United States; ^4^Department of Internal Medicine, Institute of Allergy, Yonsei University College of Medicine, Seoul, South Korea; ^5^Department of Microbiology and Immunology, University of Oklahoma, Health Sciences Center, Oklahoma City, OK, United States; ^6^Pure MHC, Oklahoma City, OK, United States; ^7^Division of Clinical Immunology, Icahn School of Medicine at Mount Sinai School of Medicine, New York, NY, United States; ^8^Department of Pediatrics, Washington University School of Medicine, St Louis, MO, United States; ^9^Department of Medicine, University of California San Diego, La Jolla, CA, United States

**Keywords:** German cockroach, T cells, allergen extract, cytokines, respiratory allergy

## Abstract

German cockroach extract is used clinically to evaluate allergen-specific sensitization and for subcutaneous allergen-specific immunotherapy, though there are no guidelines for standardization in its manufacture. We performed an immunological evaluation of 12 different cockroach extracts prepared from different sources and their potency to induce allergen-specific T cell reactivity. PBMC from 13 cockroach allergic donors were expanded *in vitro* with 12 different German cockroach extracts. After culture expansion, cells were re-stimulated with the different extracts and T cell responses were assessed by FluoroSpot (IL-5, IFNγ and IL-10 production). In parallel to the extracts, single allergen peptide pools for allergens from groups 1, 2, 4, 5, and 11 were tested to determine allergen immunodominance. Furthermore, to assess allergy specificity, PBMC from 13 non-allergic donors were also tested with the most potent extract and T cell responses were compared to the allergic cohort. Dramatic variations in T cell reactivity were observed to the different cockroach extract batches. Response magnitudes varied over 3 logs within a single donor. IL-5 production in the allergic cohort was significantly higher compared to the non-allergic cohort (p=0.004). Allergen content determination by ELISA detected much lower concentrations of Bla g 5 compared to Bla g 1 and 2. Mass spectrometric analysis revealed that Bla g 5 was present in similar amounts to Bla g 1 and 2 in extracts made from whole body, whereas it was not detected in extracts made from fecal matter, suggesting that Bla g 5 is not excreted into feces. Different donors exhibit different response patterns to different extracts, potentially dependent on the donor-specific T cell allergen immunodominance pattern and the allergen content of the extract tested. These findings have dramatic implications for the selection of potent extracts used for diagnostic purposes or allergen-specific immunotherapy.

## Introduction

German Cockroach (*Blattella germanica*) extract is used for allergy diagnosis and cockroach-specific immunotherapy (1). However, there are no guidelines regarding the standardization of its manufacture and as a result, the extracts can vary significantly with respect to allergen content. In recent years, our understanding of cockroach allergen components has grown significantly leading to a more comprehensive definition of IgE-reactive Bla g proteins (2–6).

Currently there are 10 different allergens derived from *Blattella germanica* (Bla g 1-9 and 11) that are registered in the World Health Organization/International Union of Immunological Societies (WHO/IUIS) list of Allergen Nomenclature database (www.allergen.org) (7). This number is still increasing as new allergenic targets are being discovered (8, 9). Structural biology studies investigating the different allergen components have reported that some cockroach allergens are likely secreted or excreted (Bla g 1, 2, and 4) while others, (such as Bla g 6, 7, and 8), are likely only released after breakdown of the dead insect body (10). In addition, some allergens are only expressed under specific circumstances: Bla g 4 (lipocalin) for example is only expressed by male cockroaches during reproductive activity (11).

Because of the lack of standardization of cockroach extract manufacture despite the complexity of cockroach allergen components, it is likely that different extracts will vary in terms of relative content of each allergen component, endotoxin content and other potentially immune-reactive components. Variability in extract composition can result from several factors such as the source material used (whole body vs. feces), cockroach gender ratios, diet and potentially other factors influencing allergen expression. Indeed, a study by Patterson et al. focused on Bla g 1 and 2 content, reported major variability of both allergens among the 24 cockroach extracts analyzed (12).

In contrast to other respiratory allergies such as house dust mite allergy, cockroach allergies are not associated with one or two dominant allergens. It has been shown that IgE reactivity to different allergens varies greatly in different patients (13, 14). Moreover, we have recently reported that IgE reactivity varies greatly with different German cockroach extract batches tested (15). In addition to IgE, type 2 T helper cells also contribute significantly to the pathology of allergy and asthma (16). We have previously reported that, similarly to IgE, the Bla g-specific T cell response was directed against many different allergens, and further, the pattern of allergen dominance was donor dependent, with T cell responses of different donors being dominated by different allergen specificities (9).

Given the use of extract for diagnosis and specific immunotherapy for German cockroach allergy, it is desirable that extracts used in the clinic are potent both in terms of IgE and T cell reactivity. Here, we analyzed 12 different German cockroach extracts, acquired from commercial sources as well as in-house produced extracts, determining their content for Bla g 1, 2, 4, 5, and 11 and their potency to induce antigen-specific cytokine production in T cells from cockroach-allergic patients.

## Materials and Methods

### Study Population and PBMC Isolation

Cohorts of 13 individuals sensitized to German cockroach and 13 non-sensitized individuals, as defined by cockroach-specific IgE titers (≥0.35 kU_A_/L considered positive) and skin prick test (≥3 mm considered positive), were studied. Donors were recruited from San Diego, CA, New York City, NY and St. Louis, MO following Institutional Review Board approval by the La Jolla Institute's Institutional Review Board (IRB protocol: VD-112-0217), Mount Sinai's Institutional Review Board (IRB protocol: GCO 13-0691) and Washington University Institutional Review Board (IRB protocol: 201305110). All subjects enrolled in this study provided written consent. Donor information is summarized in Table 1. IgE-titers were determined from plasma using ImmunoCAP (Thermo Fisher, Uppsala, Sweden). PBMCs were isolated from whole blood by density gradient centrifugation according to manufacturers’ instructions (Ficoll-Hypaque, Amersham Biosciences, Uppsala, Sweden).

**Table 1:** Clinical information for donor cohorts.

**Donor ID**	**CR sIgE (kU_**A**_/L)**	**Skin test (mm)**	**Clinical symptoms**
1445	76.20	9.0	Severe Asthma
1277	66.20	10.0	Intermittent Asthma
1228	56.50	7.0	Moderate Asthma
1424	45.20	10.0	Severe Asthma
1425	36.00	7.0	Severe Asthma
1398	10.50	8.5	Moderate Asthma
2210	10.13	NT	Allergic Rhinitis
1437	8.32	9.0	Mild Asthma
1175	7.27	3.0	Allergic Rhinitis
1406	5.30	4.5	Intermittent Asthma
1864	4.82	NT	Asthma
1231	4.47	3.5	Severe Asthma
2083	3.41	NT	Allergic Rhinitis
1083	<0.35	0	None
1086	<0.35	0	None
1087	<0.35	0	None
1128	<0.35	0.5	None
1190	<0.35	1	None
1199	<0.35	0	None
1200	<0.35	0	None
1204	<0.35	0	None
1225	<0.35	0	None
1227	<0.35	0	None
1235	<0.35	0	None
1242	<0.35	0	None
1283	<0.35	0	None

*NT, not tested*.

### German Cockroach Extracts

Twelve different German cockroach extracts acquired or prepared in-house were used for this study. A summary of all 12 extracts, their origin and source material as well as endotoxin content is given in Table 2. Nine of the 12 extracts were purchased from Greer Laboratories (Lenoir, NC, USA). Batches from Greer included four extracts labeled for clinical use in humans, two extracts for veterinarian use and three extracts for research use. In addition, three different research grade extracts were manufactured by different research labs. Two different extracts from German cockroach fecal matter were manufactured at Yonsei University (Seoul, South Korea), and at the La Jolla Institute (La Jolla, CA, USA), using established protocols described elsewhere (8). A third extract was manufactured from cockroach frass (cockroach debris containing body parts, fecal material and egg casings) at Indoor Biotechnologies, Inc. (Charlottesville, VA, USA) by stirring German cockroach frass for 24-48 hours at 4°C in Phosphate Buffered Saline (PBS), pH 7.4 (0.19g frass/ml). The extract was centrifuged two times and the resulting supernatant was centrifuged before filtering through a Whatman #1 filter paper and dialyzing in PBS twice. Finally, the extract was filtered through a 0.22 μm filter unit. Protein concentration for all twelve extracts was measured using Advanced Protein Assay (APA) (Cytoskeleton Inc., Denver, CO, USA), following kit instructions provided by the manufacturer.

**Table 2:** German cockroach extract summary showing origin of manufacture, lot numbers, source material, licensed use and Endotoxin content.

**Extract**	**Manufacture**	**Item #**	**Lot #**	**Source material**	**Licensed use**	**Endotoxin (EU/ml)**
Extract 1	Greer	Se05171001-01	n.a.	Whole body	Human	1,617
Extract 2	Greer	XGB46X1A5	294547	Whole body	Human	10,892
Extract 3	Greer	XGB46X1A10	294546	Whole body	Human	3,175
Extract 4	Greer	XGB46X1A10	294545	Whole body	Human	13,418
Extract 5	Greer	XB46X1A50	294544	Whole body	Veterinary	467
Extract 6	Greer	XGB46X1A50	294548	Whole body	Veterinary	3,069
Extract 7	Greer	XPB46D3A4	285969	Whole body	Research	1,889
Extract 8	Greer	XPB46D3A4	259066	Whole body	Research	1,546
Extract 9	Greer	XPB46D3A4	297511	Whole body	Research	13,782
Extract 10	In-house	n.a.	n.a.	Feces	Research	342,622
Extract 11	In-house	n.a.	n.a.	Feces	Research	152,713
Extract 12	In-house	n.a.	n.a.	Feces/Frass	Research	39,934

*n.a.-not applicable*.

### Allergen Content Determination by Mass Spectrometry

Bla g allergens were measured by a trypsin digest LCMS proteomics workflow. Extracts were digested with trypsin using the In-solution Tryptic digestion kit (Thermo Scientific, Waltham, MA, USA). Briefly, 10 μg of whole extract protein was heat denatured, reduced and alkylated with IAA for 2 h, and finally digested with trypsin for 3 h at 37°C followed by 24 h at 30°C according to manufacturer's instructions. Digests were desalted with Pierce C18 10 μl pipette tips (Thermo Scientific). Desalted digests were reduced to dryness and resuspended in 10% v/v acetic acid containing iRT standard peptides (Biognosys, Schlieren, Switzerland). All samples were digested in 4 independent experiments.

Digests were injected and analyzed by nano-LCMS with an Eksigent nanoLC400/Sciex 5600 TripleTOF system as previously described (17, 18). To obtain the tryptic peptide sequences *de novo*, one set of trypsin digests was injected and analyzed using data-dependent acquisition. The resulting spectra were interpreted using PEAKS 8.0 at a 1% FDR. Data was searched against all publicly available protein sequences from NCBInr, Uniprot, TrEMBL for the species *B. germanica* [6973].

These data were exported to mzXML by PEAKS for use in Skyline and a fragment spectral library was generated in Skyline 3. Abundant tryptic peptides from Bla g 1, Bla g 2, and Bla g 5 were selected for quantitation. Tryptic peptides and the respective transitions used for analysis are summarized in Supplemental Table 1.

For the quantitation experiments, the remaining 3 sets of digests were injected and analyzed using data-independent acquisition (DIA) or SWATH. The DIA method consisted of 37 experiments per cycle and had a total cycle time of 4.4 s. The first experiment was an MS1 survey scan from 300 to 1,500 m/z. This was followed by 36 MS/MS experiments from 350 to 1,250 m/z with 25 m/z transmission windows each. DIA data were analyzed using Skyline 3. Three isotopic precursor ions and the top 4 most abundant product ions (transitions) were selected for each tryptic peptide (Supplemental Table 1). Retention times were predicted using the iRT standard peptides and transitions were searched with an extraction window of 5 min of the predicted retention time. Initial transition peak bounders were manually confirmed. If no peak was detected for a Bla g peptide, no intensity value was reported. The sum of the peak area for the 4 product ions for all the peptides in the Bla g protein was used for quantitation of the Bla g protein. To account for LCMS variations, areas between samples were normalized to the sum peak area of the iRT peptide transitions.

### Allergen Content Determination by ELISA

Levels of allergens Bla g 1, Bla g 2, and Bla g 5 were measured by immunoassays. A two-site ELISA was performed using an allergen-specific monoclonal antibody and a polyclonal antibody for detection as described previously (19, 20).

### Endotoxin Content Determination and Removal

Each cockroach extract was analyzed for endotoxin content as previously published (15) by using the chromogenic *Limulus* Amoebocyte Lysate assay (LAL QCL kit) (Lonza, Basel, Switzerland) following manufacturer’s guidelines. The extracts were analyzed at a starting concentration of 1:100 with three 1:5 dilutions up to 1:12,500, and results were reported in endotoxin units (EU) per milliliter of extract.

Endotoxin was removed from extract 7, by passing it 3 times over a Pierce High-Capacity Endotoxin Removal Spin Column, 1 ml size (ThermoFisher). The extract was divided in half and 3 ml each was processed over 2 individual spin columns. The columns were regenerated and washed as described by the manufacturer between each column run. The final samples were assayed for endotoxin levels using a Lonza LAL QCL assay kit as described above.

### PBMC *in vitro* Cultures

For *in vitro* cultures, PBMCs were stimulated with either German cockroach extracts (10 μg/ml) or Bla g peptide pools (1 μg/ml). Cells were cultured at a density of 2 × 10^6^ cells per mL of RPMI 1640 (Omega Scientific, Tarzana, CA, USA) supplemented with 5% human AB serum (GemCell; Gemini Bio-Products, West Sacramento, CA, USA) in a 24-well plate. Cells we incubated at 37°C with 5% CO_2_. Every 3 days after initial stimulation, half of the culture volume (1 ml) was removed and 1 ml of fresh medium with IL-2 (10 U/mL; ThermoFisher) was added. After 14 days, cells were harvested and screened for IL-5, IFNγ, and IL-10 production by Fluorospot.

### Fluorospot

IL-5, IFNγ, and IL-10 production after cockroach extract stimulation was analyzed by Fluorospot assay containing three fluorophores. Flat-bottom 96-well plates with a low-fluorescent PVDF membrane (Millipore, Bedford, MA, USA) were prepared and coated with 5 μg/ml anti-human IL-5 (clone TRFK5; Mabtech, Cincinnati, OH, USA), 5 μg/ml anti-human IFNγ (clone 1-D1K; Mabtech), and 10 μg/ml anti-human IL-10 (clone 9D7; Mabtech) according to manufacturer’s instructions. Cells (1x10^5^ cells/well) stimulated with cockroach extracts were incubated with the corresponding extract (titrated at 50, 10, 2, and 0.4 μg/ml), Bla g peptide pools (1 μg/ml), medium alone and PHA (10 μg/ml) as negative and positive controls, respectively. Cells stimulated with Bla g peptide pools were incubated with corresponding Bla g pool (1 μg/ml), all cockroach extracts (10 μg/ml), medium alone and PHA (10 μg/ml) as a controls. After 24 h at 37°C, cells were removed and the plates were incubated at room temperature with detection antibodies for IL-5 diluted at 1:200 (mAb 5A10-BAM; Mabtech), IFNγ diluted at 1:200 (mAb 7-B6-1-FS-FITC; Mabtech), and biotinylated IL-10 Ab at 2 μg/ml (Clone 12G8; Mabtech). After 2 h, plates were developed by adding fluorophore conjugates for IL-5 (anti-BAM-640; Mabtech), IFNγ (anti-FITC-490; Mabtech), and IL-10 (SA-500; Mabtech) diluted at 1:200. Plates were then treated with Fluorescence enhancer-II (Mabtech) after 1 h for 15 min. Spot forming cells (SFC) were counted by computer assisted image analysis (AID *i*Spot ELR07IFL reader; Strasberg, Germany).

### Statistical Analysis

For the raw data analysis of the T cell responses as measured by Fluorospot, each reading was performed in triplicate for statistical significance. To determine if a measured response is significant, we used the following criteria, as previously published (21).

Criteria for positivity were as follows: (1) A minimum response of ≥100 SFCs per 10^6^ PBMCs. (2) The T cell reactivity values measured in triplicate in response to a given extract needed to be significantly higher (*p* < 0.05) compared to background, as assessed by Student *T*-test, two-tailed, non-parametric. The T cell reactivity observed needed to reach a stimulation index ≥2, i.e. have a magnitude at least 2-fold higher than the background. Finally, cytokine production in response to medium alone (background levels) were subtracted from all data for each stimulus.

Statistical analysis comparing T cell responses between allergic and non-allergic individuals in response to extract 7 was performed by Mann-Whitney test, non-parametric, two-tailed.

## Results

### Different Extracts Vary in Bla g Components and Endotoxin Content

The impact of varying content of German cockroach extracts on the potency to induce T cell responses has thus far not been addressed in the literature. Here, we analyzed the allergen composition and endotoxin content of 12 different extracts produced by different protocols using either whole body or cockroach fecal matter as source material.

**Figure 1:**
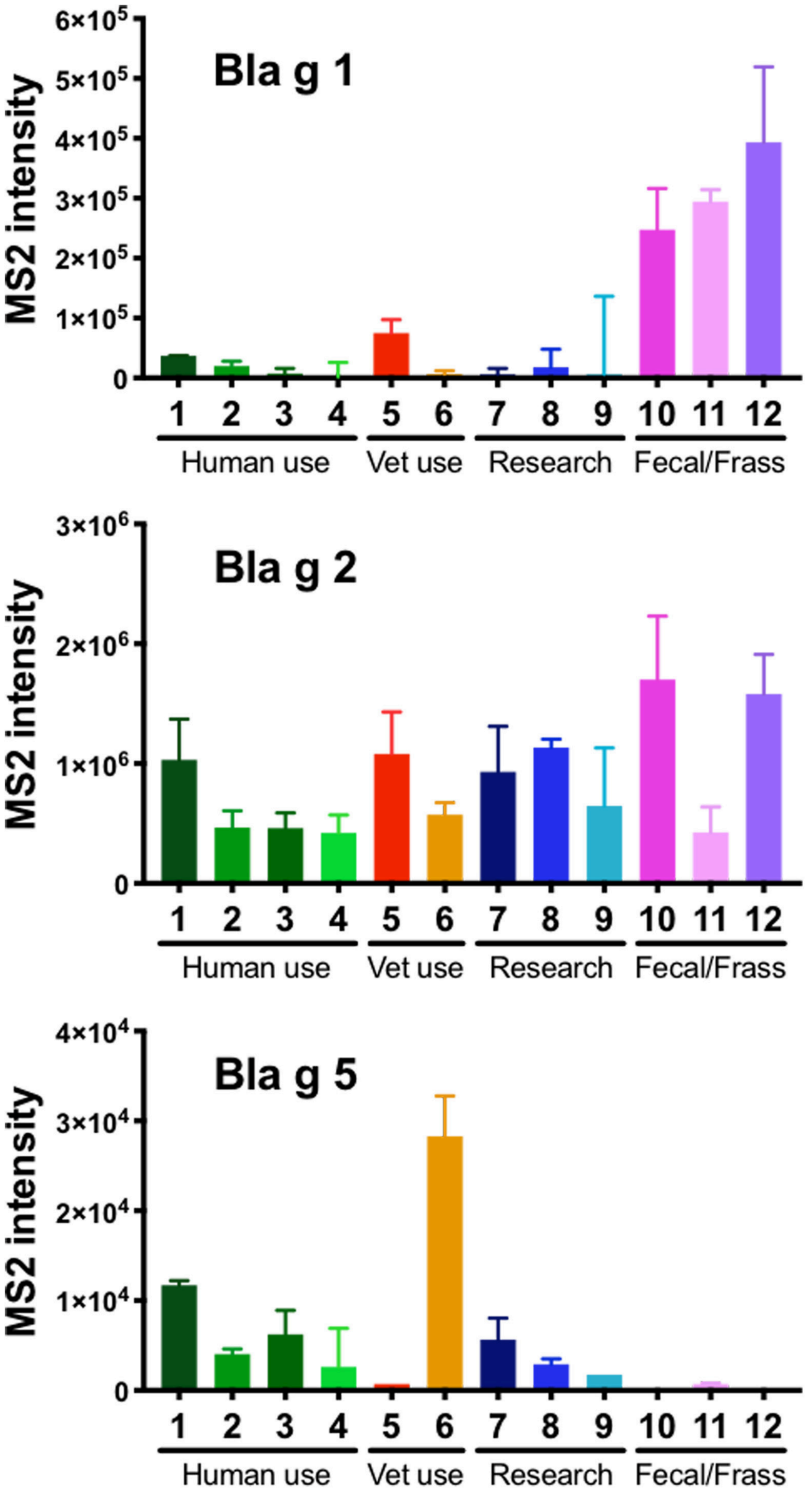
Quantification of Bla g 1, 2, and 5 in 12 different German cockroach extracts by mass spectrometry (MS). Bar graphs showing the content of peptides derived from Bla g 1, 2, and 5 as detected by MS in 12 different extracts, including extracts for human use, veterinary use, research use and extracts made from German cockroach fecal matter/frass. Bars indicate median values of triplicate readings and error bars indicate 95% confidence interval. Of note, all 3 graphs have a different y-axis.

Measurements of the relative levels of Bla g 1, 2, and 5 revealed high variability for all 3 allergens across the 12 different extracts (Figure 1). While Bla g 1 was detected in all extracts, it was observed on average 8.5-fold higher in the extracts made from German cockroach fecal matter compared to extracts made from whole body. Less variability was observed for Bla g 2, which was detected in every extract at levels within one order of magnitude of each other. Bla g 5 levels were overall much lower in comparison to Bla g 1 and 2 but were detected to some level in 10 out of 12 extracts (Figure 1). Specifically, the three fecal extracts contained little to no Bla g 5, while the extracts used in humans or for research consistently contained low amounts of Bla g 5. Interestingly, one of the extracts used in a veterinary setting contained almost 3 fold more Bla g 5 than the next highest extract (Figure 1).

In addition to mass spectrometry, we also quantified the amounts of immunoreactive Bla g 1, 2, and 5 by ELISA (Supplemental Figure 1). Overall, the contents observed were similar to the MS analysis. Interestingly, ELISA analysis detected amounts of Bla g 1 and Bla g 2 100-fold higher compared to the amounts of Bla g 5 (Supplemental Figure 1). This apparent discrepancy may indicate that the antibody-based detection of conformational epitopes requires correctly folded intact proteins and is less likely to detect degraded fragments from Bla g 5.

Assessment of endotoxin levels also revealed high variability ranging from 467 EU/ml in extract 5 (manufactured for veterinarian use) to 342,622 EU/ml in extract 10, which was made from fecal matter in-house (Table 2). Overall, endotoxin levels were highest in the extracts produced from fecal matter but even when only considering extracts labeled for human clinical use, endotoxin levels still ranged from 1,617 to 13,418 EU/ml (Table 2).

### Different Extracts Vary in T Cell Potency

Next, we set out to investigate the potency of the different extracts in terms of inducing T cell responses in PBMC from German cockroach-allergic patients.

**Figure 2:**
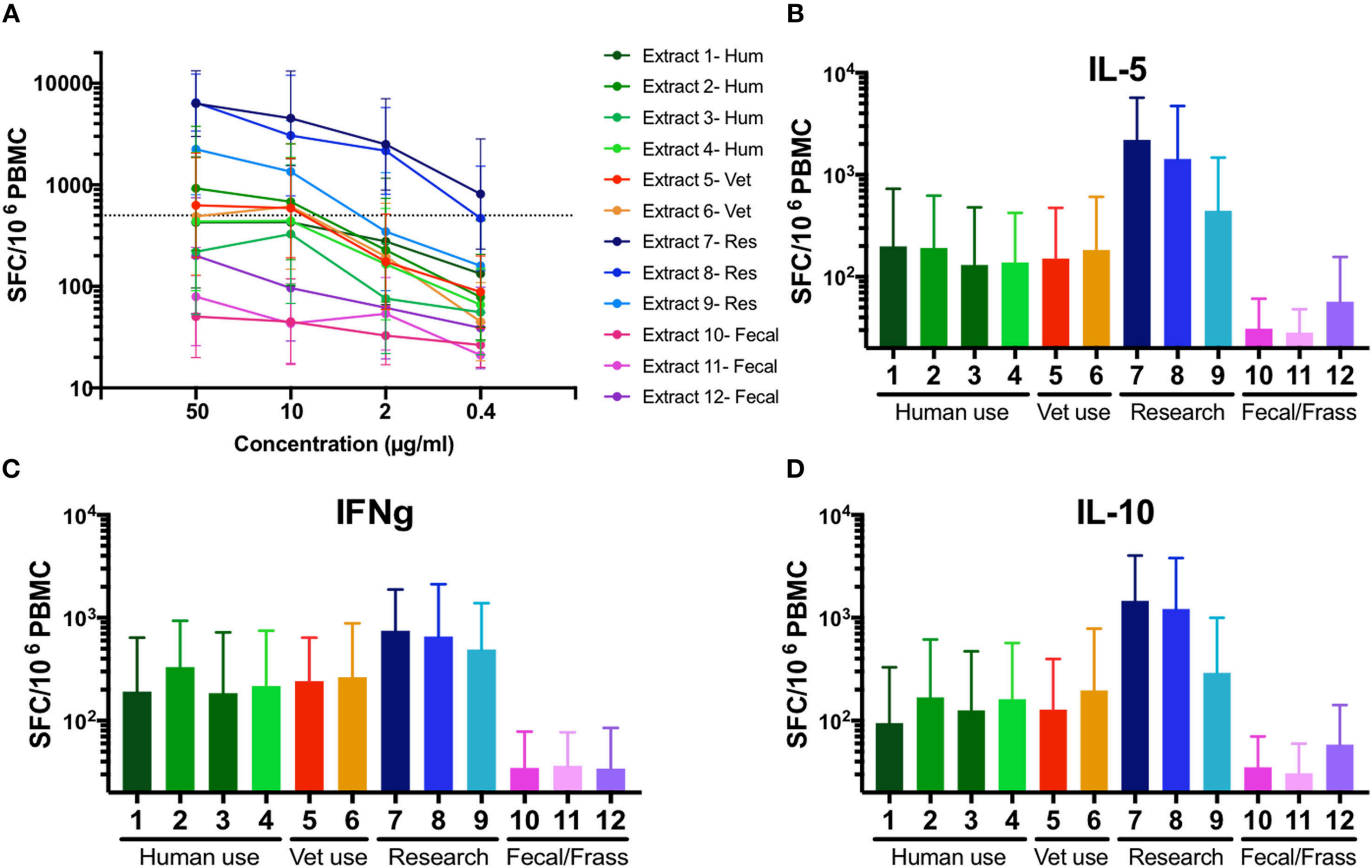
Variability in T cell potency and cytokine polarization in 12 different German cockroach extracts. **(A)** A line graph showing T cell reactivity (sum of IL-5, IFNγ, and IL-10 production) in response to 12 different preparations of German cockroach extract, measured in 5-fold titration steps (0.4, 2, 10, and 50 μg/ml). A dotted line indicates potency of extract to induce a T cell response of 500 spot forming cells (SFC). Geometric means 13 tested donors are shown, with error bars indicated 95% confidence interval (CI). **(B–D)** Bar graphs showing the IL-5, IFNγ, and IL-10 production. Bars indicate geometric means of 13 donors, with error bars indicating 95% CI. *N* = 13.

At a concentration of 50 μg/ml, the total T cell response magnitude (sum of three cytokines) ranged from 50 to 6304 SFC (extract 10, extract 7, respectively). Differences in potency are effectively visualized by dose titrations, indicating the respective allergen extract concentration required to induce a T cell reactivity of 500 SFC (Figure 2A). Spontaneous secretion of cytokines after 14 days of culture in the absence of additional extract stimulation was also assessed and the data is presented in Supplemental Table 2. Overall, the different extracts varied in potency for T cells over 2 log-fold.

Commercial extracts produced for research use were highly potent, inducing a T cell response of 500 SFC at ~4 μg/ml (extract 9) or even ≤0.4 μg/ml (extract 7 and 8) whereas extracts made for human or veterinarian clinical use required a dose of 8–50 μg/ml to induce a comparable T cell response. Extracts made from fecal material performed the worst (Figure 2A), unable to induce the 500 SFC threshold response at a concentration of 50 μg/ml. Extract 12 induced a maximum T cell reactivity of ~200 SFC and extracts 10 and 11 were unable to induce a response above 100 SFC at any tested concentration. Interestingly, no major differences in cytokine polarization were observed between IL-5, IFNγ, and IL-10 production (Figures 2B–D).

**Figure 3:**
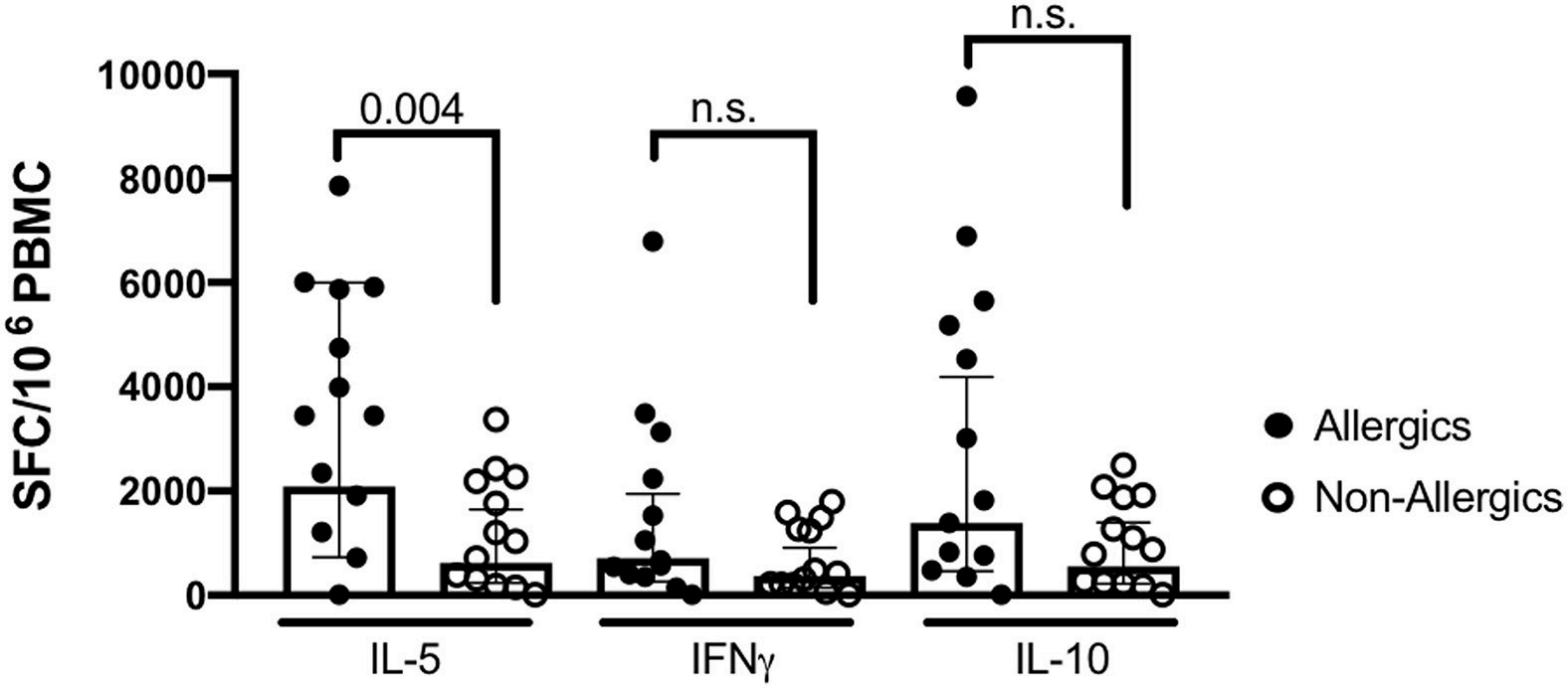
T cell reactivity measured as spot producing cells (SFC) in allergic (black dots) and non-allergic (open circles) individuals in response to extract 7. Bars indicate geometric means, error bars represent 95% confidence intervals. Statistical comparison was performed by Mann-Whitney test, two-tailed. *P* < 0.05 is considered significant. *N* = 13 per cohort.

To evaluate if the responses were allergy-specific, we performed an analysis comparing T cell reactivity (as assessed by Fluorospot) in CR allergic and non-allergic individuals, using the most potent extract (extract 7) as stimulus (Figure 3). The analysis revealed significantly stronger IL-5 production in the allergic compared to the non-allergic cohort (median responses: allergic cohort 3443 SFC vs. non-allergic cohort 1040 SFC, *p* = 0.004). No significant difference was observed for IFNγ (median responses: allergic cohort 407 SFC vs. non-allergic cohort 210 SFC, *p* = 0.21) and IL-10 (median responses: allergic cohort 1820 SFC vs. non-allergic cohort 883 SFC, *p* = 0.093).

To assess if endotoxin levels in the extracts directly affect their ability to induce T cell reactivity, we performed a correlation analysis. Endotoxin levels in the 12 extracts have been previously reported (15) and are summarized in this manuscript in Table 2. No correlation between endotoxin content and T cell reactivity was detected, regardless of whether IL-5 production alone was considered (*R*^2^ = 0.19, *p* = 0.16) or total cytokine production (sum of IL-5, IFNγ and IL-10) (*R*^2^ = 0.2, *p* = 0.16) (Supplemental Figures 2A,B). As a second approach, we also tested extract 7, for which we had a large volume available, before and after endotoxin removal. T cell reactivity was virtually the same in response to the extract before (1889 EU/ml) and after (177 EU/ml) endotoxin removal (Supplemental Figure 2C).

### Different Donors Exhibit Varying Bla g Allergen Dominance at the T Cell Level

While studies on IgE reactivity to individual German cockroach allergens have reported a wide variability of allergen recognition (13), Bla g 2 was dominant in several studies (13, 22, 23). Previous studies indicated a different pattern of dominance for T cells, with Bla g 5 being most dominantly recognized (9).

**Figure 4:**
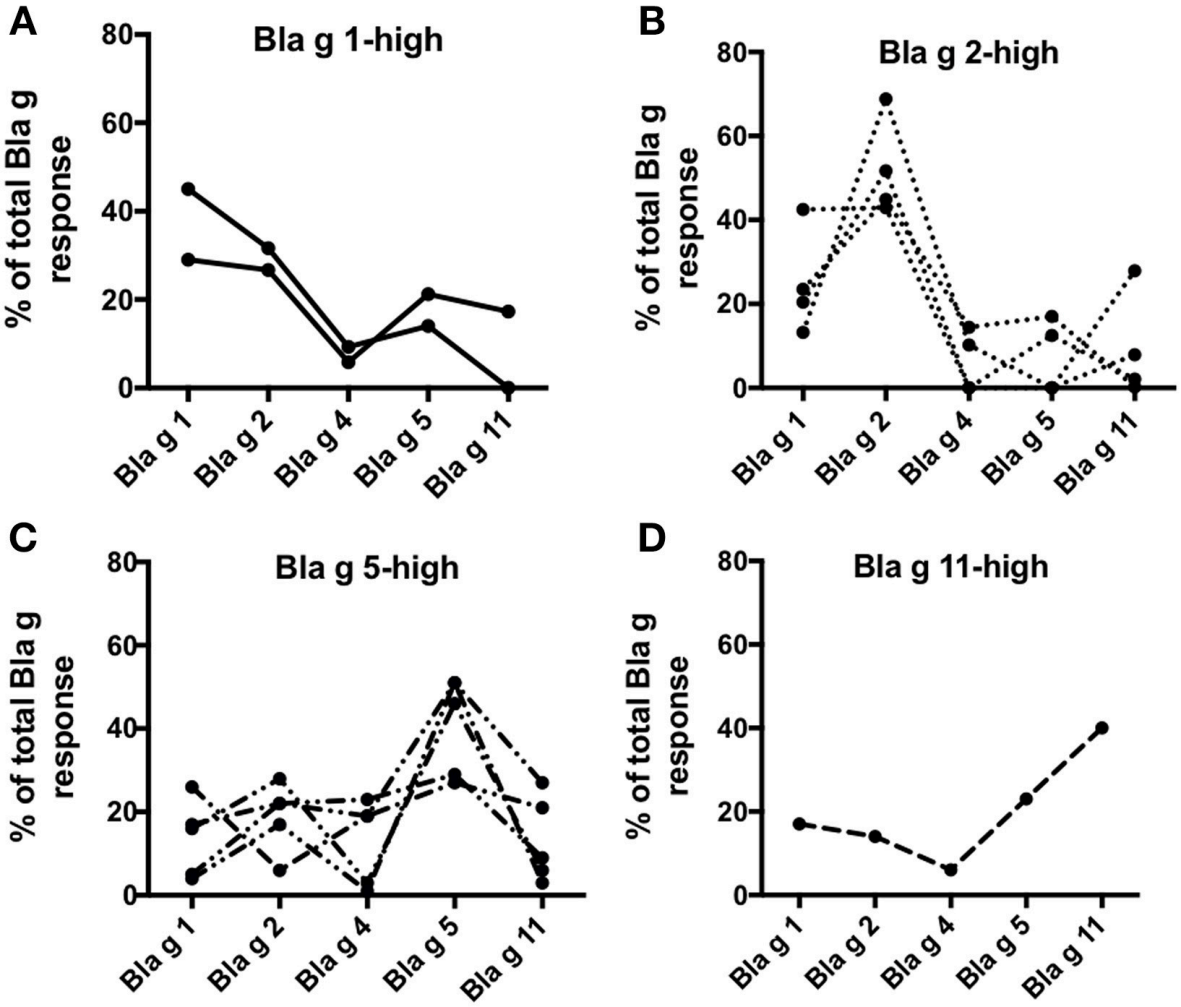
Percent T cell reactivity to Bla g-derived peptide pools. Graphs showing T cell reactivity (sum of IL-5, IFNγ, and IL-10) in individual donors against each Bla g component peptide pool as percent of the sum of all Bla g pools. Donors are grouped based on their predominant T cell reactivity for **(A)** Bla g 1, **(B)** Bla g 2, **(C)** Bla g 5, and **(D)** Bla g 11. *N* = 12.

Here we defined the patterns of Bla g allergen T cell immunodominance at the individual donor level. As expected, different patterns of dominance were noted in the different subjects. Two donors responded predominantly to Bla g 1 (Figure 4A), four donors exhibited Bla g 2-dominant T cell reactivity (Figure 4B), five donors had highest T cell reactivity in response to the Bla g 5 pool (Figure 4C) and one donor reacted most strongly to Bla g 11 (Figure 4D). One donor did not have a clear allergen-specific T cell response dominance (data not shown). This data suggests that, similar to IgE (13), allergen T cell dominance in cockroach allergy is variable as a function of the specific subject considered.

### Discordant Ranking of German Cockroach Allergic Donors as a Function of Extract Use

Given this variability in extract potency and the heterogeneity of Bla g dominance among our cohort, we anticipated that different extracts would perform differently when used to measure German cockroach extract-specific T cell responses in different donors. To exemplify this point, we selected two extracts, one with high and one with low T cell potency (extract 7 and extract 12, respectively), as previously determined (Figure 2A).

**Figure 5:**
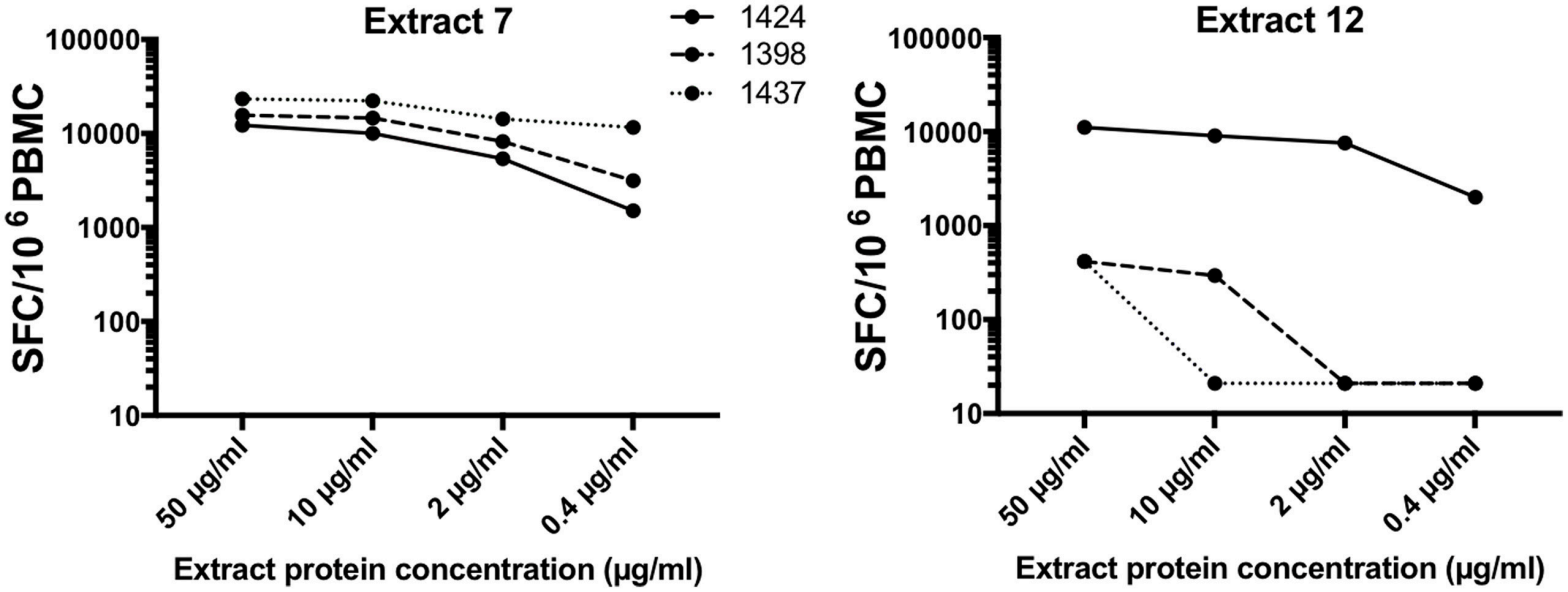
T cell reactivity in three donors in response to two different extracts. Total T cell reactivity (sum of IL-5, IFNγ and IL-10) measured as spot forming cells (SFC) in response to two selected extracts (extract 7 left; extract 12 right) in three individual donors was assessed.

The potency of these 2 extracts was assessed in 3 individual donors with different Bla g T cell dominance, namely 1424 (Bla g 1 high), 1398 (Bla g 2 high) and 1437 (Bla g 5 high). Interestingly, ranking of the three individual donors based on T cell response magnitude returns different result depending on which extract is used (Figure 5). Extract 7 elicits highest T cell responses in donor 1437 (23,267 SFC at 50 μg/ml), followed by 1398 (15,637 SFC at 50 μg/ml) and then 1424 (12,270 SFC at 50 μg/ml). In contrast, extract 12 yields the exact opposite ranking (1437- 414 SFC, 1398- 414 SFC, 1424- 11,130 SFC at 50 μg/ml). Furthermore, while T cell reactivity against extract 7 is overall high in all donors, extract 12 only elicits robust T cell responses in donor 1424 (Figure 5).

### Extract Potency Is Associated With the Donor-Specific Allergen Immunodominance and Variation in Extract Composition

To evaluate if variability in allergen content in different extracts and heterogeneity in allergen dominance among our study cohort would explain the disconnect in donor-specific T cell reactivity patterns between different extracts, we performed additional analyses. As above, we focused on extract 7 and 12 as examples of strong and weak inducers of T cell reactivity, and donors 1424, 1398, and 1437 as representatives of heterogeneous allergen reactivity.

**Figure 6:**
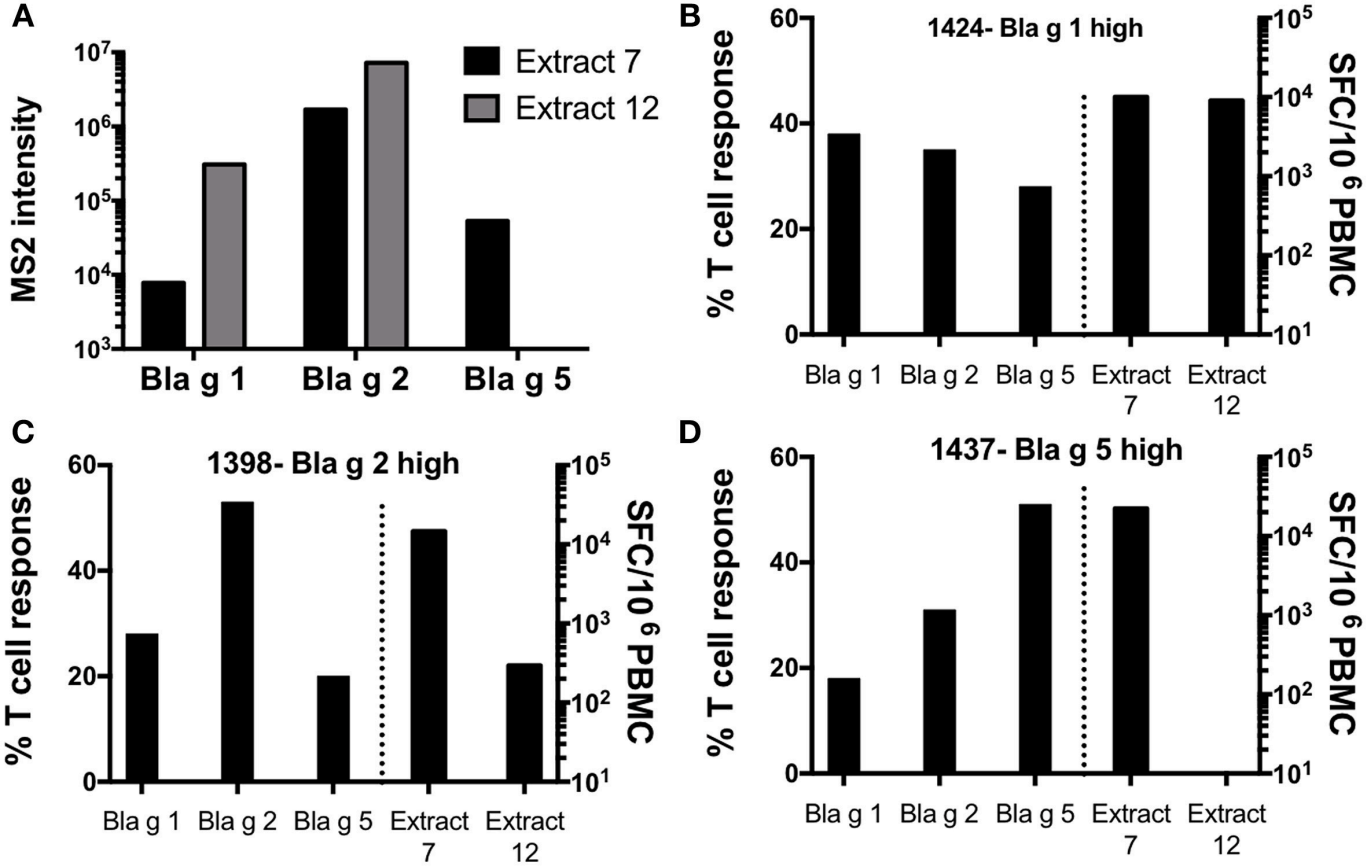
Content of Bla g 1, 2, and 5 and T cell reactivity in response to two selected German cockroach extracts. **(A)** Content of Bla g 1, 2, and 5 in extract 7 and 12 as measured by mass spectrometry. **(B–D)** Total T cell reactivity (sum of IL-5, IFNγ and IL-10) against Bla g 1, 2, and 5 -specific peptide pools and extracts 7 and 12 in three different donors, each dominant for either **(B)** Bla g 1 (donor 1424), **(C)** Bla g 2 (donor 1398) or **(D)** Bla g 5 (donor 1437). Bla g pool responses and extract responses are segregated by a dashed line. The left axis applies to the Bla g pool responses (left panel), showing percent of T cell response attributed to each individual Bla g pool. The right axis applies to the extract responses (right panel), showing T cell response magnitude as measured by spot forming cells (SFC).

Analysis of the allergen content revealed that extract 7 contains detectable levels of Bla g 1 (7,770 MS2), 2 (1,690,000 MS2), and 5 (53,100 MS2), while extract 12 contains higher levels of Bla g 1 (308,000 MS2) and 2 (7,220,000 MS2) but no Bla g 5 (Figure 6A). Both extracts induced potent T cell reactivity in donor 1424 (extract 7: 10,0057 SFC; extract 12: 8,983 SFC) (Figure 6B), who exhibits high Bla g 1 reactivity (38% of total allergen-specific response) and also reacts to Bla g 2 (35%) and 5 (28%). Similarly, donor 1398, who is associated with dominant Bla g 2 responses (53% of total allergen-specific response), reacted to both extracts (extract 7: 14,643 SFC; extract 12: 294 SFC), though reactivity to extract 7 was almost 50 fold higher (Figure 6C). Most strikingly, Bla g 5-dominant donor 1437 (51% of total allergen-specific response attributed to Bla g 5) only reacted to extract 7 (22,270 SFC) and no reactivity was observed to extract 12 (Figure 6D). This data highlights that the allergen content of a given extract and the allergen-specific T cell reactivity pattern in a given donor can have a major impact on the apparent extract’s potency to measure German cockroach-specific T cell responses, and the apparent relative reactivity of different donors might be impacted as a consequence.

## Discussion

Here we report that different CR extracts vary drastically in composition. We further show that this variation has biological consequence for measurements of T cell responses. Specifically, this can lead to paradoxically opposite ranking of T cell reactivity in individual patients. This issue is of high importance, as it can lead to potentially misleading results and should be considered during the selection of extracts to be used as a diagnostic tool, and maybe more importantly for clinical allergen-specific immunotherapy, and in the interpretation of immunotherapy results.

We investigated allergen composition and T cell potency of twelve different German cockroach extracts. Determination of allergen content by mass spectrometry and ELISA revealed high variability in Bla g 1, 2, and 5 content. This data is in line with previous reports for cockroach extracts (12) and other major allergen sources including dog and house dust mite (24, 25), reporting high variability in the content of different allergens in extracts as determined by antibody reactivity.

Antibody-based approaches to measure allergen content are reliable to determine the amount of allergen able to bind the antibody. However, any degree of protein degradation or denaturation that may alter or destroy the specific epitope recognized by the antibody will ultimately lead to the protein no longer being detected. This is an important consideration, especially with respect to T cells, that recognize short peptides and will still react to fragments of the allergen that may be present but are no longer detectable by ELISA. Interestingly, Bla g 5 was 100-fold lower in the ELISA indicating that it may be more degraded and detected less by IgG compared to Bla g 1 and 2. These results suggest that both quantitation approaches have value, and should perhaps be used in combination.

In addition to the variability in allergen content, an analysis of the potency of the different extracts tested to induce T cell reactivity revealed strong differences. Overall, commercially available extracts produced from whole body for research use induced strongest T cell responses, whereas extracts made in-house from fecal matter were weak T cell stimuli. Extracts approved for clinical use in humans performed better than extracts made from fecal matter but were several fold less potent compared to those produced for research purposes. Structural studies have reported that while some allergens, such as Bla g 1, 2, and 4, are excreted into the feces, others, particularly those with structural functions such as Bla g 6 and 7 are likely to be released after decomposition of dead bodies (10). Therefore differences in the extract source material as well as preparation will likely have a strong impact on the final composition and its immunological potency.

A potential further source of variability are the antigen presenting cells (APC). In our assay, we did not control the number of antigen presenting cells after 14 days. Different extracts may stimulate APC activation and proliferation to varying degrees, thus adding another potential source of variability for T cell reactivity. Interestingly, there was no direct effect of endotoxin content on T cell reactivity in our analysis. This may be linked to the fact that T cells themselves do not express toll like receptors that would directly interact with endotoxin. Nonetheless, it is highly possible that other immunological events not directly measured here, such as APC activation, is highly influenced by endotoxin content.

Interestingly, when comparing T cell reactivity in allergic and non-allergic individuals in response to the most potent extract, a significant difference in IL-5 production was detected, indicating that the extract does maintain its specificity to elicit allergic responses. In line with other studies (26, 27), we observed no difference in IFNγ and IL-10 production, suggesting that non-allergic individuals are not oblivious to environmental allergens but they do not exhibit a type 2 dominated T cell response as is commonly observed in allergic individuals.

Allergen sources that contain several different allergens such as German cockroach or Timothy grass are often associated with different patterns of component-resolved T cell reactivity, depending on the individual patient (9, 21). An investigation of any potential immunodominance patterns in our cohort demonstrated that no consistent pattern is observed for Bla g 1, 2, 4, 5, and 11, confirming that the responses are very heterogeneous. The resulting unpredictability of which Bla g is prominently recognized by which donor further highlights the importance of regulating the allergen content on a single component level for German cockroach extract, or in a pragmatic sense, ensuring that all allergens are well represented in the extract of choice.

Assaying T cell reactivity in a set of patients can have drastically different outcomes depending on which extract is used, as the potency of a given extract can vary greatly depending on which patient is analyzed. Our data suggests that extract potency in a given patient can be directly linked to the allergen most dominantly recognized by that patient and the content of that allergen in the tested extract. This can be of consequence in the immunotherapy setting. For example it is possible that an extract high in Blag 5 but low/negative in Bla g 2 content might be most impactful in modulating T cell responses of a donor associated with high Bla g 5 reactivity and less Bla g 2 reactivity.

It is tempting to speculate that the considerations reported here may extend to other common allergen systems. As mentioned above, extract variability has been reported for other allergen extracts (24, 25).

Taken together, these data suggest that standardized extract manufacturing procedures, in parallel with characterization of extract potency by a combination of methods, including mass spectroscopy analyses and evaluation of IgE and T cell reactivity against specific components will be informative and of significant clinical benefit.

## Ethics Statement

Review boards: La Jolla Institute’s Institutional Review Board (IRB protocol: VD-112-0217), Mount Sinai’s Institutional Review Board (IRB protocol: GCO 13-0691) and Washington University Institutional Review Board (IRB protocol: 201305110). Consent procedure: all participants provided written consent. No vulnerable populations were involved.

## Author Contributions

GB performed all T cell focused experimental work and made extract. AP, JG, and SF made in-house extract and performed allergen quantification in extract by ELISA and total protein and endotoxin content quantification of all extracts. CS provided material for extract manufacture. KJ provided inhouse made extract. CM, TV, and WH performed mass spectrometric analysis of the extracts and their allergen content. PB, AB, and LB provided clinical samples. AF and BP provided input for data analysis and in drafting of the manuscript. AS and VS designed the work, performed data analysis and interpretation and wrote the manuscript. All authors contributed to the final draft and provided approval of this manuscript.

### Conflict of Interest Statement

AP, JG, and SF are employed by Indoor Biotechnologies, Inc. The remaining authors declare that the research was conducted in the absence of any commercial or financial relationships that could be construed as a potential conflict of interest.

## Supplementary Material

Supplemental Figure 1 Quantification of Bla g 1, 2, and 5 in 12 different German cockroach extracts by ELISA. Bar graphs showing the content of Bla g 1, 2, and 5 in 12 different extracts as determined by ELISA. Of note, all 3 graphs have a different y-axis.

Supplemental Figure 2 Analysis of the effect of LPS levels on T cell reactivity. Correlations of IL-5 production **(A)** or total cytokine production (sum of IL-5, IFNγ, and IL-10) **(B)** expressed as spot-forming cells (SFC) in response to a given extract and their corresponding LPS content. **(C)** A graph showing T cell reactivity (sum of IL-5, IFNγ, and IL-10 production) in response to Extract 7 (licensed for human use) before and after endotoxin removal (177 vs. 1889 EU/ml), measured in 5-fold titration steps (0.4–50 μg/ml). A dotted line indicates potency of extract to induce a T cell response of 500 spot forming cells (SFC).

Supplemental Table 1 Mass spec anaysis of 12 difffernt german cockroach extracts.

Supplemental Table 2 Levels of spontaneous cytkine release at day 14 in the absence of additional extract stimulation.
