# Supplementary figures and images for: Variability in German Cockroach Extract Composition Greatly Impacts T Cell Potency in Cockroach-Allergic Donors
Source: Front Immunol. 2019 Feb 27;10:313. doi: 10.3389/fimmu.2019.00313 (PMC6413722; doi:10.3389/fimmu.2019.00313)

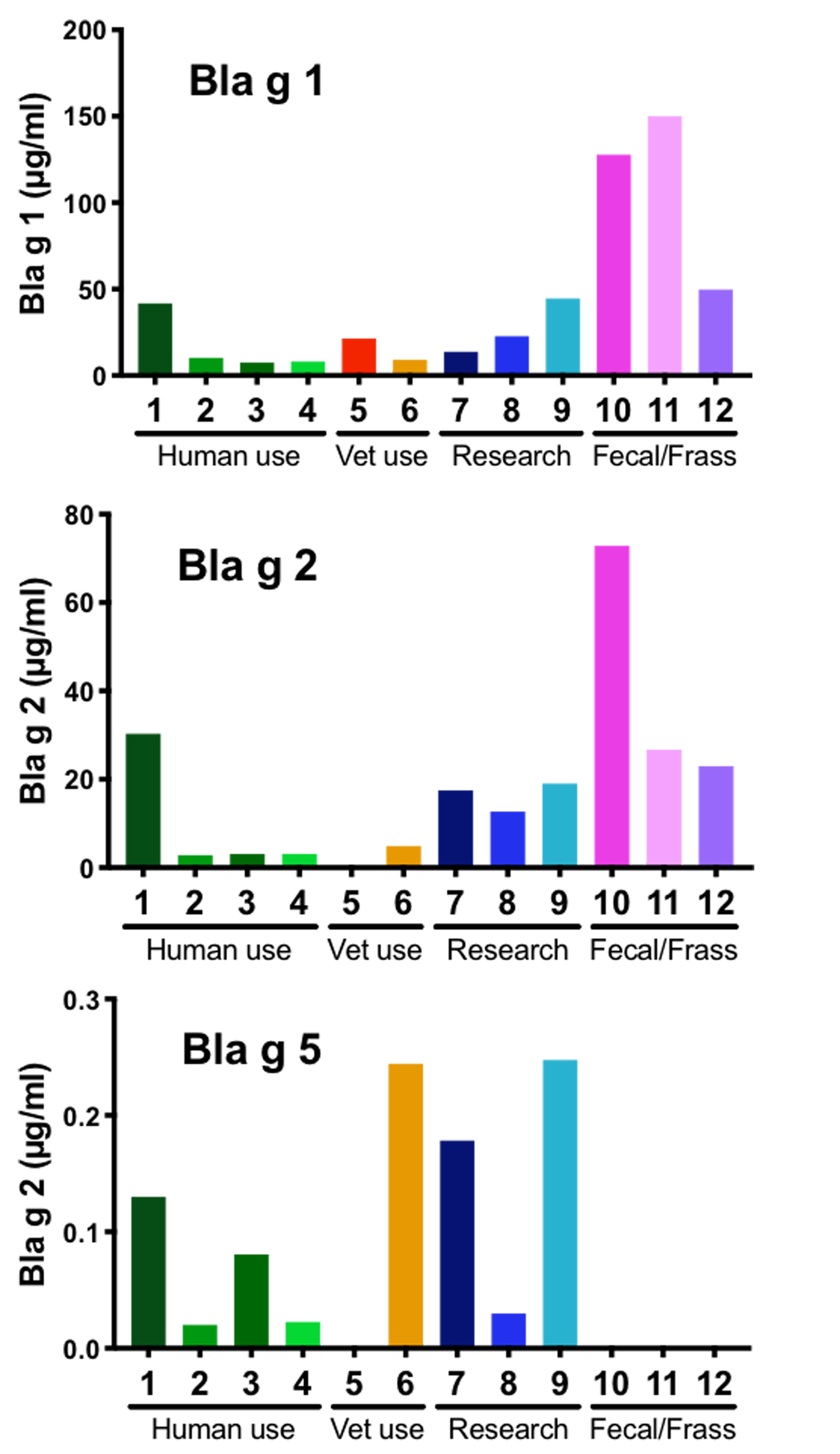

Supplement: Supplemental Figure 1 — Quantification of Bla g 1, 2, and 5 in 12 different German cockroach extracts by ELISA. Bar graphs showing the content of Bla g 1, 2, and 5 in 12 different extracts as determined by ELISA. Of note, all 3 graphs have a different y-axis. [file Image_1.TIF]

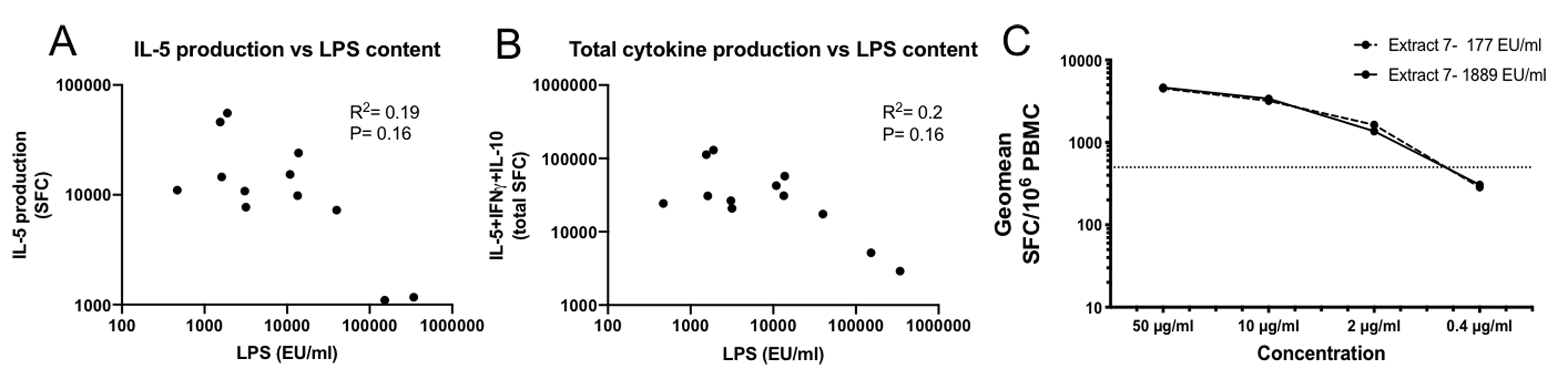

Supplement: Supplemental Figure 2 — Analysis of the effect of LPS levels on T cell reactivity. Correlations of IL-5 production (A) or total cytokine production (sum of IL-5, IFNγ, and IL-10) (B) expressed as spot-forming cells (SFC) in response to a given extract and their corresponding LPS content. (C) A graph showing T cell reactivity (sum of IL-5, IFNγ, and IL-10 production) in response to Extract 7 (licensed for human use) before and after endotoxin removal (177 vs. 1889 EU/ml), measured in 5-fold titration steps (0.4–50 μg/ml). A dotted line indicates potency of extract to induce a T cell response of 500 spot forming cells (SFC). [file Image_2.TIF]
